# The Prevention and Cure of Cholera Infantum—So-Called

**Published:** 1883-04

**Authors:** 


### THE PREVENTION AND CURE OF CHOLERA INFANTUM—SO-CALLED.

We shall not offer any apology to our readers for appropriating so much space in this issue to the article by Dr. Cumming on Artificial Human Milk, at the expense of the usual variety of practical matter which it has been our habit to lay before them, with each recurring number of this journal. For, a careful perusal of this contribution to one of the most important subjects that can possibly engage the attention of the medical profession, will convince our friends that, in presenting in detail the views of our esteemed contributor, we have rendered them and the cause of humanity a real and permanent service.

It is proper to state that the article in question is not published now for the first time. A portion of it appeared a number of years ago in book form, and a portion has already been published in a medical journal. The two parts are now combined, rearranged, and laid before the profession in its present shape, in the confident belief that it will reach many persons to whom it has heretofore been inaccessible, and that adherence to the principles herein so clearly declared will result in the saving of many little lives every year.

The simplicity of style, which the author has attempted, and which he has so successfully attained, was the result of an original intention, on his part, of making his little book not only useful to medical men, but available as a guide to mothers and nurses as well; nevertheless, it is believed that the article will challenge the most rigid scrutiny, and survive the most exacting scientific investigation.

We do not aspire to the distinction of being considered an enthusiast, but in making the claim for the system advocated in this paper,—the claim indicated in the above caption,—we know whereof we affirm, and, after several years of attentive trial, are persuaded that in this system of feeding infants we have the surest and best means of not only preventing but actually curing a very large percentage of those cases of gastric, intestinal and gastro-intestinal catarrh (?), commonly collectively denominated "cholera infantum." Experience has taught us that often even in apparently extreme cases of the uncomplicated affection, the strict and faithful observance of these simple rules will suffice to secure amendment, and at most the addition of merely a digestive tonic, as pepsine and bismuth, is the only medication required.

We think we hazard nothing in asserting if all of the proprietary "infants' foods," all of the farinaceous preparations so frequently recommended and so generally employed, and all of the opiates and astringents so lavishly administered, could be assigned to a common grave, and this plan substituted for the erroneous and prevalent practices of the present day, that our annual mortality reports would not thereafter indicate such a slaughter of the innocents, and the living reward would be reaped by unbroken family circles, in happy homes all over the land.

We not only commend this admirable paper to the careful attention of all practitioners, but more especially to lecturers and writers on the diseases of infancy.

